# Effects of Covid-19 Vaccination during Pregnancy on the Obstetric and Neonatal Outcomes in a Tertiary Health Care Center

**DOI:** 10.34763/jmotherandchild.20232701.d-22-00043

**Published:** 2023-07-06

**Authors:** Gargee Suman Tripathy, Tanushree Sandipta Rath, Saujanya Behera, K Shruti Lekha, Dattatreya Kar, Sujata Pendyala

**Affiliations:** Department of Obstetrics and Gynaecology, IMS & SUM Hospital, Siksha O Anusandhan (Deemed to be) University, Bhubaneswar - 751003, Odisha, India; Department of Paediatrics, Veer Surendra Sai Institute of Medical Sciences and Research (VIMSAR), Burla, Sambalpur, Odisha, India; AIPH (Asian Institute of Public Health) University, Bhubaneswar, Odisha, India; Department of Medical Research, IMS & SUM Hospital, Siksha O Anusandhan (Deemed to be) University, Bhubaneswar - 751003, Odisha, India

**Keywords:** COVID-19 vaccines, pregnancy, obstetric outcomes, neonatal outcomes

## Abstract

**Background:**

Pregnancy is an immuno-compromised state, and pregnant women with COVID-19 are at an increased risk for adverse pregnancy outcomes. Thus, the Center for Disease Control and Prevention (CDC) and the Advisory Committee on Immunization (ACIP) have advocated for COVID-19 vaccination in pregnant women. COVAXIN and COVISHIELD were the vaccines being used in India in the first phase of vaccination, but limited data exist on pregnancy outcomes regarding SARS-CoV-2 vaccines and pregnancy and lactation.

**Material and methods:**

A retrospective study was conducted which included only women who delivered after 24 weeks gestation. Women with an unknown vaccination status or with past or active COVID-19 infection were excluded. Demographic characteristics, maternal and obstetric outcomes, and fetal and neonatal outcomes were compared between the unvaccinated and vaccinated groups. Statistical analysis was done with Chi-square testing and the Fisher exact test using SPSS-26 software.

**Results:**

Deliveries before a gestation of 37 weeks were significantly higher in the unvaccinated group compared to the vaccinated group. Rates of vaginal deliveries and preterm deliveries were found to be higher in the unvaccinated population. Women who had taken COVAXIN had a higher rate of adverse events compared to those who had taken COVISHIELD.

**Conclusion:**

There were no significant differences in adverse obstetric outcomes attributed to vaccine administration between the vaccinated and unvaccinated pregnant women. The beneficial effects of the vaccines in protecting against COVID-19 infection, particularly in pregnancy, outweigh the minor adverse events associated with vaccine administration.

## Introduction

Pregnancy is an immuno-compromised state. Though pregnant women are not more prone to contract COVID-19 than the general population, pregnancy tends to alter the body’s immune system, which can occasionally be related to more severe symptoms in the case of COVID-19 infection [1,2]. The cardiopulmonary changes of pregnancy like diaphragm elevation, increased oxygen consumption, hyperventilation, and respiratory tract edema make pregnancy more prone to a hypoxic state, and as such, certain articles and studies suggest an increased susceptibility of COVID-19 infection in pregnant women [3,4,5]. Another aspect suggesting this is the fact that SARS-CoV-2 uses the protein Angiotensin-Converting Enzyme (ACE2) as a receptor to invade cells, and these receptors are extensively found in the placenta and fetus, making the placenta a target for infection and possibility for vertical transmission [6,7]. Furthermore, pregnant women infected with COVID-19 might be at an increased risk for adverse pregnancy outcomes, such as preterm birth, compared to pregnant women without COVID-19 [8]. Thus, the Center for Disease Control and Prevention (CDC) and the Advisory Committee on Immunization (ACIP), in collaboration with the American College of Obstetricians and Gynecologists and the American Academy of Pediatrics, have issued guidelines indicating that COVID-19 vaccines should not be withheld from pregnant women [9]. Most pregnant patients required medical attention during their third trimester; therefore, vaccination was proposed prior to the 30th week of gestation [10]. Moreover, women who are infected with SARS-CoV-2 in their first trimester may be at an increased risk of a miscarriage [11]. COVID-19 vaccines can be offered at any gestational age in pregnancy [12]. However, safety data on COVID-19 vaccination during pregnancy remain limited [13]. The first COVID-19 vaccines available in the US were messenger RNA (mRNA) vaccines: BNT162b2 (Pfizer–BioNTech) and mRNA1273 (Moderna) [14]. There are several COVID-19 vaccines validated for use by the WHO (given Emergency Use Listing). As of 12 January 2022, vaccines that have obtained EUL (Emergency Use Listing) are the Pfizer/BioNTech Comirnaty vaccine, the SII/COVISHIELD and AstraZeneca/AZD1222 vaccines, the Janssen/Ad26.COV 2.S vaccine developed by Johnson & Johnson, the Moderna COVID-19 vaccine (mRNA 1273), the Sinopharm COVID-19 vaccine, the Sinovac-CoronaVac vaccine, the Bharat Biotech BBV152 COVAXIN vaccine, the Covovax (NVX-CoV2373) vaccine, and the Nuvaxovid (NVX-CoV2373) vaccine [15].

The two vaccines that were being used in India in the first phase of vaccination, COVAXIN and COVISHIELD, will be considered in this study. COVAXIN, India^’^s indigenous COVID-19 vaccine by Bharat Biotech, was developed in collaboration with the Indian Council of Medical Research (ICMR) and National Institute of Virology (NIV). This inactivated vaccine was developed using Whole-Virion Inactivated Vero Cell derived platform technology. The COVISHIELD vaccine, developed by The Serum Institute of India Pvt Ltd., is made from a weakened version of an adenovirus. This vaccine is an Indian version of the AstraZeneca COVID-19 vaccine. To date, limited data exist on vaccine and pregnancy outcomes relating to SARS-CoV-2 vaccines in pregnancy and lactation. This study was conducted to analyze pregnancy outcomes in COVID-19-vaccinated women as compared to those who are not vaccinated against COVID-19.

## Materials and methods

This is a retrospective study conducted from 1 October 2021 to 31 December 2021, at IMS and SUM Hospital, Bhubaneswar, which included all women with singleton pregnancy who delivered there after 24 weeks of gestational age. Women with an unknown vaccination status and women with past or presently active COVID-19 infection were excluded from the study. All data were collected from the labour room of the IMS and SUM hospital. Demographic characteristics, maternal and obstetric outcomes, and fetal and short-term neonatal outcomes were compared between the unvaccinated and vaccinated group. Statistical analysis was done with a Chi-square test and a Fisher exact test using SPSS-26 software. A p value of <0.05 was considered significant.

Amongst the 14 women excluded from the study, 2 women were excluded due to their unknown vaccination status which could not be collected from their records. The other 12 women had active COVID-19 infection at the time of admission. Of the 12 COVID-19 positive cases, 7 women were unvaccinated, whereas 5 women had received at least one dose of COVID-19 vaccine during pregnancy.

**Figure 1.**
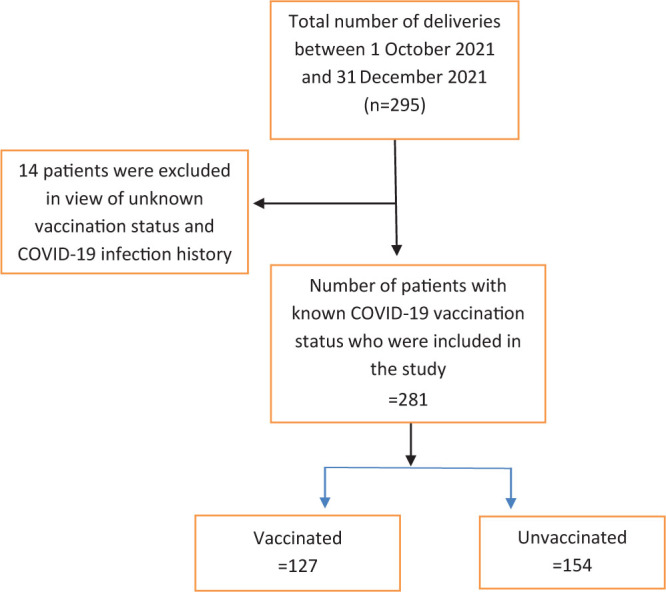
Vaccination status in the study group of patients.

## Results

A total of 295 women delivered at IMS & SUM Hospital between 1 October 2021 and 31 December 2021. After the exclusion of 14 patients due to unknown vaccination status or being infected with COVID-19, the total number of study participants was 281. Out of the 281 participants, 127 (45.19%) had received at least 1 dose of COVID-19 vaccine, while 154 (54.80%) participants were unvaccinated. Out of the vaccinated study participants, 57 (44.88%) women had taken the COVISHIELD vaccine while 70 (55.11%) had taken COVAXIN.

Comparisons were done with respect to demographic and obstetric characteristics between the groups of women who had received at least 1 dose of vaccine (vaccinated) and those who did not receive any vaccine (unvaccinated). This data is presented in Table 1. Maternal age in years at the time of delivery (28.24 ± 4.1 vs 28.12 ± 4.6; p value = 0.81), gravidity, and parity were comparable. Average gestational age at the time of delivery was found to be significantly higher in the unvaccinated group (avg= 37.6 ± 2.4, p = 0.033).

**Table 1.** Demographic and Obstetric Parameters

**Parameters**	**Unvaccinated N=154**	**Vaccinated N=127**	**p value**
Age	28.12 ± 4.6	28.24 ± 4.1	0.81
Gravidity			
<3	121 (78.57)	107 (84.25)	
≥3	33 (21.43)	20 (15.75)	0.29
Parity			
0	108 (70.12)	89 (70.07)	
1	40 (25.97)	34 (26.77)	
2	5 (3.24)	4 (3.14)	
3	1 (0.64)	0 (0)	0.839
Abortion			
≤1	142 (92.20)	117 (92.12)	
>2	12 (7.80)	10 (7.88)	0.561
Gestational age at delivery	37.6 ± 2.4	37.1 ± 2.1	**0.033**
Multifetal gestation	3 (1.94)	6 (4.72)	0.704
Previous LSCS	17 (11.03)	15 (11.81)	0.839

Individual obstetric and maternal outcomes are presented in Table 2. Adverse maternal outcomes were compared between the two groups. Deliveries at less than 34 weeks gestation were higher in the unvaccinated population as compared to the vaccinated group, but the change was statistically not significant. Deliveries at less than 37 weeks gestation (preterm deliveries) were also significantly higher in the unvaccinated population as compared to the vaccinated group. However, the average gestational age at delivery was marginally higher in the unvaccinated group as compared to the vaccinated group (37.6±2.4 vs 37.1±2.1). Furthermore, the rate of vaginal delivery was found to be higher amongst the unvaccinated population as compared to the vaccinated group.

**Table 2.** Maternal and Obstetric Outcomes

**Parameters**	**Unvaccinated N=154**	**Vaccinated N=127**	**p value**
Gestational age at delivery	37.6 ± 2.4	37.1 ± 2.1	0.033
Gestational age at delivery <34 weeks	7 (4.54%)	6 (4.72%)	0.07
Gestational age at delivery <37 weeks	33 (21.42%)	17 (13.28%)	0.001
Vaginal delivery	63 (40.90%)	48 (37.79%)	0.041
Caesarean delivery	91 (59.09%)	79 (62.20%)	
Anaemia (Hb <7gm/dl)	8 (5.19%)	6 (4.72%)	0.495
Gestational + pregestational Diabetes	10 (6.49%)	4 (3.14%)	0.2
Hypertension in pregnancy	9 (5.84%)	10 (7.87%)	0.555
PPROM	8 (5.19%)	11 (8.66%)	0.43
Hypothyroidism	17 (11.03%)	17 (13.38%)	0.548

Table 3 shows the fetal and neonatal outcomes such as intrauterine growth restriction, intrauterine fetal death, preterm deliveries and NICU admissions following delivery among both the groups. Preterm deliveries were found to be significantly higher in the unvaccinated group (p value- 0.001).

**Table 3.** Fetal and Neonatal Outcomes

	**Unvaccinated N=154**	**Vaccinated N=127**	**p value**
SGA	8 (5.19%)	6 (4.72%)	0.369
IUFD	5 (3.24%)	2 (1.57%)	0.469
PRETERM	33 (21.42%)	17 (13.28%)	**0.001**
NICU admissions	28 (18.18%)	25 (19.68%)	0.749

The APGAR score is comprised of 5 components: colour, heart rate, reflexes, muscle tone, and respiration, each scored 0, 1, or 2. This is an accepted method for reporting the status of a newborn and the response to resuscitation when needed. It is reported at 1 minute and 5 minutes after birth. A score of 7–10 at 5 minutes is considered reassuring. After excluding 7 stillbirth babies, the APGAR score was compared between the vaccinated and unvaccinated populations among the 274 live births in Table 4.

**Table 4.** APGAR Scores

**APGAR Scores**	**Unvaccinated (Live births=149)**	**Vaccinated (Live births=125)**	**p value**
1 min score <7	9 (6.04%)	15 (12%)	0.66
5 min score <7	2 (1.34%)	5 (4%)	

In Table 5, any kind of adverse reactions following vaccination with the two kinds of COVID-19 vaccines, in terms of fever, myalgia, vomiting, injection site pain, and headache, are shown. It was observed that, out of all the women who had taken COVAXIN, 30% (21 women out of 70) reported adverse reactions, which was higher compared to those who had taken COVISHIELD (22.8%; 13 women out of 57).

**Table 5.** Adverse Reactions Associated with COVID-19 Vaccines

**Symptoms**	**Covaxin**	**Covishield**
Fever	3	5
Headache	9	2
Injection site pain	1	0
Myalgia	6	5
Vomiting	2	1
TOTAL	21 (30%)	13 (22.8%)

In the study population out of the 127 vaccinated women, 27 women (21.25%) received at least one dose of vaccine in their first trimester, 51 women (40.15%) in their second trimester, and 49 women (38.58%) in their third trimester. In Table 6, we have compared the fetal and maternal outcomes of the vaccinated population based on the time of vaccination.

This study found no significant difference between the three compared groups of vaccinated women receiving COVID-19 vaccine in their first, second and third trimesters respectively, when compared in terms of maternal outcomes (gestational and pregestational diabetes, hypertension in pregnancy, preterm prelabour rupture of membranes, and anemia) and fetal outcomes (NICU admissions and preterm birth).

**Table 6.** Fetal and Maternal Outcomes vs Time of Vaccination

**Criteria**	**1^st^ Trimester Vaccination (N=27)**	**2^nd^ Trimester Vaccination (N=51)**	**3^rd^ Trimester Vaccination (N=49)**	**Total**	**p value**
NICU admissions	2	9	14	25	0.07
Preterm birth	3	8	6	17	0.81
Gestational and pregestational diabetes	0	1	3	4	0.56
Hypertension in pregnancy	2	5	3	10	0.78
PPROM	4	3	4	11	0.41
Anemia	3	2	1	6	0.19

## Discussion

This study evaluates the effects of COVID-19 vaccination during pregnancy on the obstetric and neonatal outcomes in a tertiary health care center. The two vaccines that were taken into consideration were COVAXIN and COVISHIELD, as both were extensively administered during the mass vaccination program rollout in India. The characteristics of the vaccinated and unvaccinated groups were comparable in all parameters. While comparing the maternal outcomes, both the vaccinated and unvaccinated groups were comparable, except for the gestational age at delivery. This study showed higher gestational age in the unvaccinated group (p = 0.033), which is in accordance with a multi-centric retrospective cohort study conducted by Rottenstreich et al. [16]. Interestingly, the unvaccinated group had an average higher gestational age at delivery, but the number of preterm deliveries was also significantly higher in this group. Multiple large retrospective studies found no significant difference in occurrence of anemia, gestational diabetes, and hypertensive disorder of pregnancy between unvaccinated and vaccinated groups, which is also similar to our results [16, 17].

Out of the 154 unvaccinated women, 63 had a normal vaginal delivery and 91 had a caesarean delivery. In the vaccinated group, out of 127 women, 48 had a normal delivery and 79 had a caesarean delivery. The difference in both the groups is insignificant (p=0.833). However, in a study by Rottenstreich et al., 712 pregnant women who received two doses of COVID-19 vaccine during the third trimester showed a significantly increased rate of elective caesarean delivery [16].

Data gathered from the internet indicated that maternal and neonatal complications were evident in pregnant women with severe COVID-19 infection [18,19,20]. In the pregnant population, COVID-19 vaccines are as effective and safe as in the general population [14,21,22,23]. As per a large retrospective cohort study, it was seen that COVID-19 infection during pregnancy was associated with a higher risk of preterm deliveries [24,25]. In our study, it was found that there was a significantly higher number of preterm deliveries amongst the unvaccinated group when compared to the vaccinated group (p=0.001), which was in accordance with a retrospective cohort study in a tertiary center [17]. However, this finding was not in accordance with available large western epidemiological studies [22,26]. In their secondary analysis, a large retrospective cohort study focusing on time of vaccination, it was found that women vaccinated in their second trimester had a significantly higher number of preterm deliveries [27]. Our study did not find any significant difference in preterm prelabour rupture of membrane between the two groups, similar to results found by Peretz et al. [17].

This study did not find any significant difference in the number of babies who were small for gestational age between the groups, and this result is consistent with available western studies [16,26,27].

Similar to our study, a few western studies also found no significant increase in intrauterine deaths among the vaccinated and unvaccinated group [13,23,26,28,29]. NICU admissions post-delivery were also comparable among both the groups [17].

COVID-19 vaccines can be offered at any gestational age in pregnancy, but the second dose should preferably be completed before the third trimester [12]. Also, no increased risk for miscarriage has been seen among pregnant women who received an mRNA COVID-19 vaccine in early pregnancy (before 20 weeks) or any increased risk of birth defects [13,30]. A study analyzing anti-spike antibodies in maternal blood and umbilical cord blood at the time of delivery found that the levels of antibodies were higher in women whose initial vaccination course was in third trimester [31]. However, antibody levels were still high and protective even for those women whose initial vaccination was early or few weeks prior to pregnancy. The present study also shows no significant difference in fetal and maternal outcomes when compared amongst the three groups of women having received a COVID-19 vaccine in their first, second or third trimester.

## Conclusion

COVID-19 vaccines should be routinely advocated for the pregnant population. There were no significant differences in adverse obstetric outcomes attributed to vaccine administration between the vaccinated and unvaccinated pregnant women. On the other hand, preterm deliveries, which carry significant adverse obstetric significance, were more common in the unvaccinated group. The beneficial effects of the vaccines in protecting against COVID-19 infection, particularly in pregnancy, outweigh the minor adverse events associated with vaccine administration.
